# Metabolomics of Oxidative Stress in Recent Studies of Endogenous and Exogenously Administered Intermediate Metabolites

**DOI:** 10.3390/ijms12106469

**Published:** 2011-09-28

**Authors:** Jia Liu, Lawrence Litt, Mark R. Segal, Mark J. S. Kelly, Jeffrey G. Pelton, Myungwon Kim

**Affiliations:** 1 Department of Anesthesia, University of California, San Francisco, CA 94143, USA; E-Mails: liujia@anesthesia.ucsf.edu (J.L.); MyungwonKims@gmail.com (M.K.); 2 Department of Epidemiology and Biostatistics, University of California, San Francisco, CA 94143, USA; E-Mail: mark@biostat.ucsf.edu; 3 Department of Pharmaceutical Chemistry, University of California, San Francisco, CA 94143, USA; E-Mail: Mark.Kelly@ucsf.edu; 4 Physical Biosciences Division, Lawrence Berkeley National Laboratory, Berkeley, CA 94720, USA; E-Mail: jgpelton@berkeley.edu

**Keywords:** metabolomics, metabonomics, oxidative stress, NMR, proteomics, transcriptionomics, multivariate analysis, Principal Component Analysis, targeted profiling, chemometrics, brain, liver, kidney, heart

## Abstract

Aerobic metabolism occurs in a background of oxygen radicals and reactive oxygen species (ROS) that originate from the incomplete reduction of molecular oxygen in electron transfer reactions. The essential role of aerobic metabolism, the generation and consumption of ATP and other high energy phosphates, sustains a balance of approximately 3000 essential human metabolites that serve not only as nutrients, but also as antioxidants, neurotransmitters, osmolytes, and participants in ligand-based and other cellular signaling. In hypoxia, ischemia, and oxidative stress, where pathological circumstances cause oxygen radicals to form at a rate greater than is possible for their consumption, changes in the composition of metabolite ensembles, or *metabolomes*, can be associated with physiological changes. *Metabolomics* and *metabonomics* are a scientific disciplines that focuse on quantifying dynamic metabolome responses, using multivariate analytical approaches derived from methods within genomics, a discipline that consolidated innovative analysis techniques for situations where the number of biomarkers (metabolites in our case) greatly exceeds the number of subjects. This review focuses on the behavior of cytosolic, mitochondrial, and redox metabolites in ameliorating or exacerbating oxidative stress. After reviewing work regarding a small number of metabolites—pyruvate, ethyl pyruvate, and fructose-1,6-bisphosphate—whose exogenous administration was found to ameliorate oxidative stress, a subsequent section reviews basic multivariate statistical methods common in metabolomics research, and their application in human and preclinical studies emphasizing oxidative stress. Particular attention is paid to new NMR spectroscopy methods in metabolomics and metabonomics. Because complex relationships connect oxidative stress to so many physiological processes, studies from different disciplines were reviewed. All, however, shared the common goal of ultimately developing “omics”-based, diagnostic tests to help influence therapies.

## 1. Introduction

The innovations produced and inspired by the Human Genome Project included new approaches to analyzing problems where the number of people or organisms in a study is enormously smaller than number of biomarkers—genes in the case of *genomics*, proteins in the case of *proteomics*, transcription factors in the case of *transcriptionomics*, and metabolites in the case of *metabolomics* and *metabonomics*. The new consolidation of fundamental intellectual concepts common to statistical analyses in all of these multivariate disciplines has been given the label of “omics”. Advances in measurement technologies that have occurred in parallel in each of the “omics” specialties have led to an explosion of advances and publications. In metabolic studies the tradition has gone from focusing on a limited number of key metabolites having principal roles in the situation under investigation, to focusing on a metabolomics or metabonomics approach. Because large numbers of accurate metabolite quantifications can now be made in many instances, it is possible that aggregates of metabolite changes might reveal mechanisms or key metabolic reactions that are not discernable in hypothesis-based studies of limited subsets. Numerous review papers have provided overviews of metabolomics and metabonomics from different mechanistic and physiological perspectives [1–13]. None of the reviews, however, have specifically emphasized the complex area of oxidative stress. Our research focus, which is the use of NMR technologies for understanding healthy and unhealthy neurophysiological situations involving oxidative stress, has motivated us to review the metabolomics and metabonomics literature with this focus. In this paper we present our views of the most important conceptual and practical points about oxidative stress and metabolomics and metabonomics, and then discuss important recent advances and studies, going from studies which emphasized a limited number of metabolites to studies that emphasized the behavior of entire *metabolomes*.

## 2. Results and Discussion

### 2.1. What is Metabolomics? What is Metabonomics?

The ensemble of metabolites in an organism is known as its *metabolome*. In 1999 the term *metabonomics* was devised to describe “the multiparametric, quantitative study of dynamic metabolome responses in living systems to physiological and pathophysiological stimulation or genetic modification” [14–16]. However, a competing term appeared in 2001: *metabolomics*, that was generally defined as: the “comprehensive and quantitative analysis of all metabolites…” [16,17]. Various publications have made distinctions between the two words so as to prescribe and proscribe situations where each term should be used. However, dictionaries created by lexicographers since Samuel Johnson, who wrote in an 18th century in a preface that “no dictionary can embalm language, and secure it from corruption and decay”, are at least in part descriptive, accepting as meanings those intended in daily use by people. Many papers have used the terms metabolomics and metabonomics interchangeably, despite assertions that such should not be done [16]. We are using the word metabolomics throughout this review to include what is in the metabonomics definition given above.

As with genomics, proteomics, and transcriptionomics, the mathematics within metabolomics developed from the Human Genome Project, a massive research effort between the mid-1990s and 2003 that produced refined multivariate analyses for situations where the number of biomarkers greatly exceeds the number of subjects. Although one can obtain data from as many as hundreds or thousands of humans or animals, the number of human genes is estimated to be 30,000 to 40,000, while the number of essential human metabolites is estimated as “more than 3,000” [18], with additional, complex metabolites possible from combinations of those essential. The general multivariate approaches, which became known as “the omics”, serve as key tools for elucidating biological mechanisms, screening for disease, stratifying patient risks, and ultimately shaping the individualization of medical therapies. In comparison to quantifications of transcription factors and proteins, metabolic quantifications more accurately reflect biological endpoints. Although the term “metabolomics” was used occasionally prior to 1995, the high output from very productive investigators and consortia at Imperial College London led to it having multiple meanings. Today “metabolomics” is: a household word, a society, a journal (Metabolomics, Springer), an FDA entity (Center for Metabolomics, Division of Systems Toxicology, NCTR) and an NIH Roadmap Initiative. Its primary methodologies for data acquisition are high-resolution ^1^H and ^13^C NMR spectroscopy and mass spectroscopy, although additional spectroscopic methodologies are also involved. Its primary methodologies for multivariate statistical analysis includes the collection of techniques known as SIMCA—Soft Independent Modeling of Class Analogy, which involves hierarchical cluster analysis, principal components analysis, different types of partial least squares analysis, and subsequent modeling with new regression algorithms.

### 2.2. Oxidative Stress Comes from Excited Electrons that Provide the Chemical Energy for Life

The metabolic energy in living systems comes mostly from harnessing excitation energy that was transferred to electrons during photochemical or chemical interactions. At the molecular level living systems capture excited electron energy in *electron transport systems (ETS)*, which for aerobic metabolism are located in inner mitochondrial membrane protein assemblies. Electron energy is harnessed along their cascade to oxygen, the ultimate electron acceptor in living cells. Except for fluorine, which is minimally present in live entities, oxygen has the highest affinity for electrons. Why do plants and animals need to use complex electron transport systems to capture energy for metabolism? The answer, no doubt, was given by Thaddeus Dobzhansky (1973) when he wrote, “Nothing in biology makes sense except in the light of evolution”. Indeed, electron transport chains for biological energy extraction are known to have been in single cell systems since early evolution, billions of years ago [19,20]. Such seems quite reasonable because of “the Photoelectric Effect”, the basic physics phenomenon in which light dislodges a bound atomic electron, giving it energy to move to a higher energy orbit or escape. Nobel Prizes in physics for understanding the Photoelectric Effect went to Heinrich Hertz, who discovered the effect in 1887, and to Albert Einstein, who fully explained it in 1905. In early evolution, when single-cell life began in the oceans, the sun’s rays, beating down relentlessly and generating unimaginably large numbers of dislodged electrons, gave full Darwinian advantage to systems that could capture some or all of the enormous chemical energy provided by the sun to excited electrons.

## 3. Reactive Oxygen Species and Oxidative Stress

The orbital properties of oxygen’s electrons account for its reactive properties as a radical. An understanding of that began with Michael Faraday’s discovery in the 1840’s that oxygen is paramagnetic (attracted into a magnet.) However, it was not until 1925, after quantum mechanics became more widely appreciated, that the mechanism was fully understood. Robert Mulliken explained it by noting that in its lowest energy state, molecular oxygen has two unpaired electrons. Each electron has an intrinsic angular momentum, or spin, of ½, meaning that in a magnetic field it has only two quantum states (a doublet): along the magnetic field and opposite to it. Together the spin angular momentum of oxygen’s two unpaired, spin ½ electrons, which are in different orbits, add up to a spin angular momentum of 1, which in quantum mechanics is a *triplet state. Triplet oxygen* is the lowest energy state of molecular oxygen, and is the normal, commonly existing state. There is also an excited orbital state in which the two normally unpaired electrons become paired in the same orbit, with one spin aligned opposite to the other, producing spin 0, which is a singlet. This excited and potent but short-lived oxidant state is known *as singlet oxygen*. A molecule with an unpaired electron is a *radical*—except for free molecular oxygen, O_2_! Radicals are highly reactive chemically. If oxygen within a molecule has an unpaired electron, that molecule is known as an *oxygen radical. Reactive oxygen species* (ROS) are chemically-reactive molecules containing oxygen, such as hydrogen peroxide (H_2_O_2_) and the hydroxyl free radical (HO·). Oxygen’s extremely high affinity for electrons can also allow it to take on an extra electron to form the *superoxide anion radical* O_2_ ^−^·, which is also an ROS molecule. Henceforth we will denote O_2_ ^−^· by the shortened term “superoxide”. In living systems there is a constant formation of oxygen radicals, along with a system of reactions that constantly consumes them. When cells or organisms produce oxygen radicals at a greater rate than they can be consumed by the intracellular systems built for that purpose, there is a condition of *oxidative stress,* and a concern that there will be harmful ROS effects.

In mitochondrial oxidative phosphorylation the final chemical reaction produced by the electron transport chain is

$$\text{NADH}+1/2{\text{O}}_{2}+{\text{H}}^{+}\Leftrightarrow {\text{H}}_{2}\text{O}+{\text{NAD}}^{+}+\text{energy}$$

Atomic oxygen collects 2 electrons here, with the basic reaction being

$$2{\text{H}}^{+}+2{\text{e}}^{-}+1/2{\text{O}}_{2}\Rightarrow {\text{H}}_{2}\text{O}+\text{energy}$$

which can be appreciated in the first equation from knowing that an early step in the electron transport chain was

$$\text{NADH}\Leftrightarrow {\text{NAD}}^{+}+{\text{H}}^{+}+2{\text{e}}^{-}$$

The “energy” produced in the reaction occurs as a proton gradient across the inner mitochondrial membrane, and is used in the phosphorylation reaction that produces ATP.

It is inevitable that the substrates in the high rate basic reaction will sometimes produce superoxide, where the reaction is

$${\text{e}}^{-}+{\text{O}}_{2}\Rightarrow {{\text{O}}_{2}}^{-}$$

Evolution has produced key antioxidant enzymes and scavengers to deal with this, the primary ones being: superoxide dismutase, which exists in different forms in mitochondria and the cytosol; catalase, which is present primarily in peroxisomes; and glutathione peroxidase, which is found both in the cytosol and in mitochondria. Superoxide dismutase catalyzes

$$2{{\text{O}}_{2}}^{-}\cdot +2{\text{H}}^{+}\Rightarrow {\text{H}}_{2}{\text{O}}_{2}+{\text{O}}_{2}$$

while catalase catalyzes

$$2{\text{H}}_{2}{\text{O}}_{2}\Rightarrow {\text{O}}_{2}+2{\text{H}}_{2}\text{O}$$

Glutathione is a tripeptide consisting of cysteine, glycine, and glutamate. It exists in reduced (GSH) and oxidized (GSSG) states and catalyzes the decomposition of H_2_O_2_ and organic peroxides:

$$\text{XOOH}+2\text{GSH}\Rightarrow \text{GSSG}+\text{XOH}+{\text{H}}_{2}\text{O}$$

Although glutathione, along with SOD are the major intracellular antioxidants, the major blood plasma antioxidants are uric acid, vitamin C (ascorbate), vitamin E (α-tocopherol), thiols, and bilirubin [21]. Uric acid is produced during purine degradation by xanthine oxidase from xanthine and hypoxanthine. Increased uric acid levels are common during hypoxia and ischemia, and are a marker for oxidative stress [21].

An important additional path for superoxide damage was appreciated after it was discovered that the messenger molecule responsible for vasodilation, initially known as EDRF (Endothelium-Dependent Relaxation Factor), could be identified as nitric oxide (NO) [22]). The nitric oxide radical NO combines with superoxide to form peroxynitrite [23].

$$\text{NO}+{{\text{O}}_{2}}^{-}\Rightarrow {\text{ONOO}}^{-}$$

which among its many reactions produces the lasting marker nitrotyrosine [24,25]. Nitric oxide together with radicals derived from its reactions with either superoxide or oxygen, are known collectively as *reactive nitrogen species* (RNS) [26]). Because they often act together, RNS are as much a concern as ROS in oxidative stress [27].

During physiological increases in metabolism, for example during exercise, increases in oxygen consumption increases the production of oxidants that can produce damage, such as that coming from muscular fatigue after strenuous exercise. Protective radical scavenging is accomplished not only by reactions just described, but also by scavenging by many other naturally present compounds that contain keto-carbonyl and/or rings, such as vitamin E, vitamin C, and many TCA Cycle metabolites. Although radical damage is the topic of this review, it should not be forgotten that excessive antioxidant activity might be disadvantageous to organisms if it interferes with mechanisms of recovery and adaptation. For example, in ischemic preconditioning, transient increases in reactive oxygen species can provide protection, which in turn can be blocked by antioxidants [28]. Given all of the above, it is easy to see that perturbations of metabolite distributions can result from oxidative stress, oxidative damage, and even from antioxidant therapy. Thus it is appropriate to use multivariate metabolomics analysis techniques to assess and categorize oxidative protection, injury, and recovery.

## 4. Investigations of Two Metabolites as Treatments in Oxidative Stress

### 4.1. Fructose-1,6-bisphosphate

**Figure 1:**
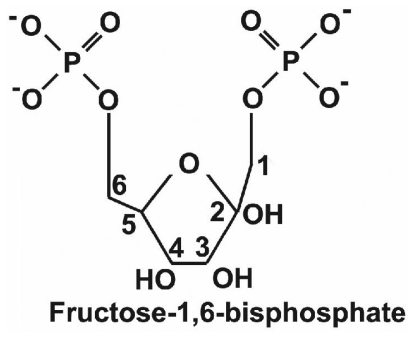
Schematic diagram of the chemical structure of Fructose-1,6-Bisphosphate (FBP). Two negatively charged phosphate groups (carbons 1 and 6) make it possible for FBP to chelate calcium.

In 1980 Dr. A.K. Markov and collaborators published the first of two decades of manuscripts written by his group and others about treating hypoxia, ischemia, or oxidative stress with exogenously administered fructose-1-6-bisphosphate (FBP, see Figure 1). In the decades before the year 2000, FBP was sometimes referred to as FDP, “fructose diphosphate”, which is inconsistent with chemistry nomenclature, as the latter requires that the phosphates be adjacent when using “diphosphate”. This point can be important when searching the literature. Markov’s early work [29,30] described *in vivo* and *in vitro* preclinical experiments in which FBP improved physiological functions and ATP preservation during and after periods of oxygen deprivation. The use of exogenous FBP was motivated by a clever idea. The authors reasoned that in hypoxia and ischemia, anaerobic glycolysis might be suppressed or shut down if there were not enough ATP available early in glycolysis for phosphorylation of fructose-6-phosphate, and/or also if the acidosis of hypoxia would, additionally, excessively inhibit the enzyme that is involved: phosphofructokinase (PFK). They reasoned that substrate entry into glycolysis of one exogenously provided FBP molecule would, compared to substrate entry of one glucose molecule, spare the expenditure of two ATP’s (spent on glucose by hexokinase and phosphofructokinase). Using FBP instead of glucose as a glycolytic substrate would thus yield four ATP’s per substrate molecule instead of two. Markov *et al*. administered FBP during different circumstances of oxygen deprivation. Dramatic improvements in myocardial function and survival were seen [30], and the general conclusion arrived at by these investigators was that “exogenous FBP will restore the activity of glycolysis, which has been inhibited by acidosis, by intervening in the Embden-Meyerhoff pathway, both as a metabolic regulator and as a high-energy substrate”. At around the same time dramatic findings were also being found by others [31] in a study where hypoxic FBP-treated rabbits breathed for an average of 21 min after the initiation of 4% FIO_2_ hypoxia, with all being resuscitated after cardiac arrest, compared to glucose treated rabbits breathing for an average of only 1.4 min, with only a ≈20% success in resuscitation after cardiac arrest. FBP was also found to be protective in focal cerebral ischemia, whether it was given both before or after occlusion, or after reperfusion [32]. As well it was found protective in hypoxia studies with astrocyte cultures [33,34] and cerebrovascular endothelial cultures [35].

Dr. Markov’s work on FBP protection has continued over many years, with publications appearing as recently as 2007 [36,37]. During the twenty years that followed his initial FBP experiments, many others, too many to report, studied FBP use in hypoxia, ischemia, and/or oxidative stress. Post-ischemic outcome improvements were found by some to be dramatic, by others to be simply modest [38–40], and by a smaller number to be completely nonexistent [41–43]. Although many important, significant, animal investigations of FBP’s protective and therapeutic actions have been carried out since the 1980’s, and although much has also been learned about molecular mechanisms of hypoxic/ischemic injury, the mechanisms by which FBP protects various tissues were never clearly established, and FBP administration never became part of routine clinical care. Still, many have maintained that the successful FBP studies posed a formidable intellectual challenge: could the improved outcomes associated with FBP administration be explained by important, already known, injurious mechanisms? For example, in brain ischemia typical mechanisms that are suggested include glutamate excitotoxicity, calcium overload, acidosis, oxygen radicals, PARP-1 activation, apoptosis. The challenging question is: are there or are there not new, important, undiscovered protective molecular mechanisms that the successful FBP treatments are trying to reveal?

Many studies have in their discussions that “alternative hypotheses of FBP protection have included calcium chelation, free-radical scavenging, and protease inhibition”. In recent years numerous studies have pointed to radical scavenging as FBP’s primary protective mechanism [44–46]. An antioxidant hypothesis fits well with other recent work that has emphasized that strong antioxidant protection is provided by the keto-carbonyl groups that are found in numerous metabolic intermediates. These are alleged to confer strong antioxidant properties via peroxidative decarboxylation, which allows α-keto acids (R-CO-COO^−^) to scavenge H_2_O_2_ radicals. For example, in the case of pyruvate, R is CH_3_, and the products of the reaction are acetate and the formate radical, with the latter easily converting to H_2_O + CO_2_. Such scavenging is preferable, for example, to superoxide removal by superoxide dismutase, which consumes two H^+^ ions that could be used by ATPase to generate ATP [47]. Interesting experiments in which DNA injury has lead to PARP-1 activation and NAD^+^ depletion, which is very bad for sustaining glycolysis, have hypothesized, and then found true, that after PARP-1 activation cells can be rescued by exogenous administration of TCA Cycle intermediates such as pyruvate and α-ketoglutarate [48,49]. In those rescue studies the motivation for administering pyruvate and α-ketoglutarate was to provide the TCA Cycle with substrates distal to glycolysis, thereby boosting ATP production in a manner reminiscent of Dr. Markov’s original motivation. Curiously, other researchers were publishing papers at the same time that the above mentioned FBP studies were being done, but where the use of α–κɛτο TCA substrates were recommended because of their antioxidant properties, not because of TCA Cycle substrate properties [50–54].

### 4.2. Pyruvate

**Figure 2:**
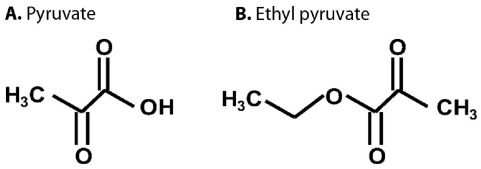
Schematic diagram of the chemical structures of pyruvate (**A**) and ethyl pyruvate (**B**). Ethyl pyruvate is formed by an ester linkage of pyruvate (right side of the structure in Figure 2B) with ethanol (left side of the structure in Figure 2B).

Pyruvate is able to directly, non-enzymatically accomplish radical scavenging, as mentioned earlier with regard to the way peroxides are neutralized. This raises the question: which mechanism is more important when rescuing cells by providing metabolic substrates that bypass glycolysis and directly feed the TCA Cycle: the metabolic help that the substrates provide in sustaining ATP, or the substrates’ antioxidant properties? For example, it is possible in experimental situations where PARP-1 activation has been proven to occur, to detect decreased NAD^+^ and decreased cytosolic glycolysis, and then perform a rescue with exogenous administration of pyruvate and other TCA Cycle substrates. In experimental investigations of this, rescue does not occur if glucose is used. With ^13^C NMR spectroscopy it is also possible, using ^13^C-enriched TCA substrates, to detect the TCA metabolism of those administered substrates that cause the rescue. To address the question just posed about which is more important, we changed the protocol of a previously completed study, and redid the experiments, so as to compare two oxidative stress rescue protocols [55]. In the first protocol we tried the rescue by adding a nonmetabolizable antioxidant radical scavenger, PBN, to the external medium. In the second protocol we added a metabolizable TCA substrate, ethyl pyruvate, to the external medium. The structures of pyruvate and ethyl pyruvate are compared in Figure 2. Ethyl pyruvate easily enters cells and efficiently delivers pyruvate to the cytosol. The result in the particular protocol and experimental situation that was studied was that the nonmetabolizable antioxidant provided the same ATP protection as the addition of the metabolic substrate. In a mouse model of Parkinson’s disease ethyl pyrvate provided neuroprotection by suppressing astroglial myeloperoxidase expression, NADPH oxidase-, and/or inducible NO-synthase- derived reactive oxygen species/reactive nitrogen species production [56]. Other evidence of the antioxidative effects of pyruvate include the inhibition by topically or orally administrated pyruvate of the formation of cataracts induced by photochemical generation of reactive oxygen species [57,58]. An NMR-based metabolomic study supported a model in which the long lifespan of slcf-1 mutants relies on increased mitochondrial pyruvate metabolism coupled to an adaptive response to oxidative stress [59]. The above experiments illustrated the potential importance of antioxidant actions by metabolites, and also suggest that the mechanism of protection and help provided by exogenously administered FBP, which does not significantly enter many types of healthy cells, might well be based in its antioxidant properties.

**Figure 3:**
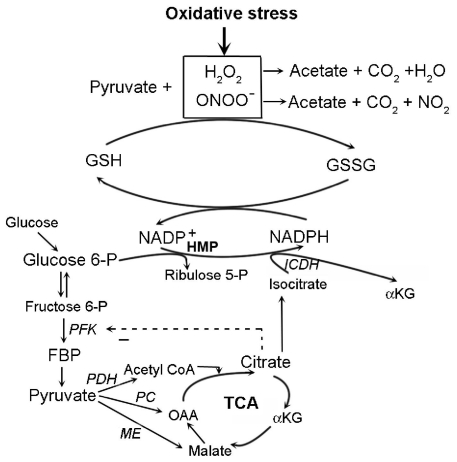
Several key reactions are shown that depict the antioxidant roles of pyruvate and FBP. Hydrogen peroxide and peroxynitrite, shown at the top of the diagram, are reactive oxygen and nitrogen species formed during oxidative stress. One antioxidant mechanism of pyruvate is associated with the keto-carbonyl group that can scavenge peroxides and peroxynitrites in direct decarboxylation reactions. The antioxidant role of keto-carbonyl groups in metabolites would help relieve the free radical burden handled by the GSH system. Pyruvate can indirectly help maintain reduced glutathione (GSH) instead of oxidized glutathione (GSSG) by increasing NADPH production. Pyruvate carboxylation by pyruvate carboxylase (PC) and/or malic enzyme (ME) increases the formation of citrate, which can suppress phosphofructokinase (PFK) activity, and drive glucose-6-phosphate into the hexose monophosphate pathway (HMP); or it can generate isocitrate, the substrate for NADP^+^-dependent isocitrate dehydrogenase (ICDH).

As was summarized in a review of heart experiments [60], pyruvate can protect via mechanisms that do not directly involve PARP-1 reactions. Excess pyruvate drives the pyruvate carboxylase (PC) and/or malic enzyme reactions towards the formation of increased mitochondrial citrate. Citrate effluxes to the cytosol where it increases NADPH formation by two mechanisms: (1) suppression of PFK activity which causes diversion of glucose-6-phosphate into PPP; and (2) generation of isocitrate substrate for converting NADP^+^ into NADPH via isocitrate dehydrogenase. Increases in NADPH and reducing equivalents bolster GSH restoration from GSSG. The increased ratio of GSH/GSSG in pyruvate-enriched cardioplegia has been found to dampen the myocardial inflammation induced by oxidative stress [61]. Figure 3 presents a schematic diagram of chemical reactions in oxidative stress. Some studies found that pyruvate has other antioxidant mechanisms, such as the up-regulation of glutathione peroxidase mRNA levels [62], the activation of different anti-apoptotic pathways [62], and suppression of mitochondria DNA damage and protein degradation [63]. By comparison, administration of lactate, which some investigators have found to be an important fuel for the brain [64–66], did not prevent brain energy depletion in a rodent model with a severe diffuse traumatic brain injury (TBI) [67]. The issue of lactate *vs*. pyruvate as an energy source for neurons has not been fully clarified. It has been conjectured that increased synaptic activity stimulates glial metabolism and production of lactate that can be consumed by neurons [64,68] but simultaneously increased neuronal metabolism of glucose and lactate is probably not possible [65,66].

## 5. The Mechanics of Metabolomics

The front end of all metabolomics projects is accurate data acquisition. All approaches work best when optimum state-of-the-art techniques are use to obtain high signal-to-noise data in which different metabolites appear precisely in the same spectral location with identical characteristics. This is often not trivial, as many NMR resonance peaks shift with pH, and LC/MS or LC/NMR peaks also shift from chromatography changes. With changes in time or circumstance, metabolites in a data set very commonly exhibit large variations in their absolute concentrations, with biological relevance sometimes being more in the percentage variation rather than in having the largest absolute value. For all of these reasons preprocessing is commonly performed in which data variables are centered and scaled with the methods deemed most appropriate in each situation. One approach (Pareto scaling) sets the mean value of each variable as the zero reference, and then scales magnitudes into standard deviation units. However, there are many other available approaches, such as logarithmic or exponential scaling [69]. Appropriate quality controls must be established by investigators for each type of data acquisition and processing.

**Figure 4:**
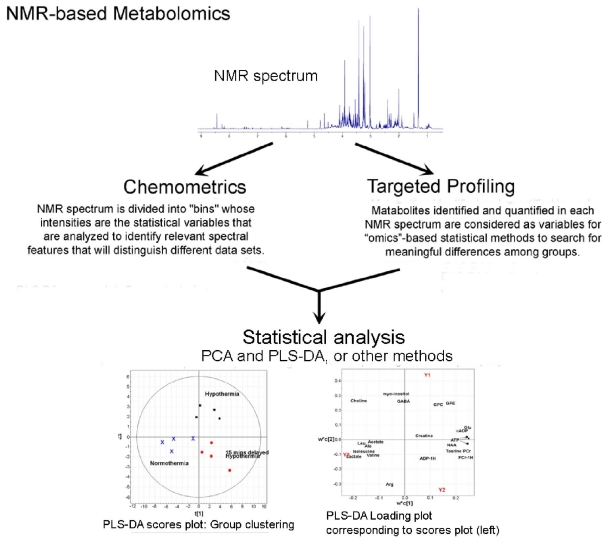
Schematic diagram showing alternate analysis paths in NMR-based metabolomics analyses. One begins with ^1^H NMR spectra similar to the one at the top of the figure, obtained at the NIH-supported Central California 900 MHz NMR Facility at UC Berkeley during a study of rat brain extracts. The ^1^H resonance for water at 4.66 ppm has been removed. The chemical shifts of most metabolites measured *in vivo* and in many studies of extracts are typically to the right of 4.66 ppm (upfield), but ^1^H nuclei on many important compounds, such as ATP and NADH, are found to the left of 4.66 ppm (downfield). In the first step of NMR metabolomics, accurate high resolution spectra such as the one shown are obtained from solutions containing processed biological fluids or tissues from clinic or laboratory sources. As explained in the text NMR spectra are then typically compared by one or both of two approaches: *chemometrics* and *targeted profiling*. In each of the approaches it is common to perform unsupervised Principal Component Analysis (PCA), and supervised Projection to Least Squares-Discriminant Analyses (PLS-DA), and each produces Scores Plots and Loading Plots, as explained in the text, and exemplified in the bottom part of the figure, where data from three treatment groups cluster apart from each other, and metabolites that led to separations are found in corresponding quadrants of the Loadings Plot.

The next step in metabolomic analyses is the application of pattern recognition algorithms that find statistical differences and attach them to distinct biological phenomena. The “omics” approach generated popularity for certain basic algorithms found in many commercial software packages. Two distinct approaches have been established for processing NMR spectra, as outlined in Figure 4. In the first, known as *chemometrics*, no attempt is made to identify specific metabolites in the spectra. Spectral patterns and intensities are statistically compared somewhat as in a fingerprint analysis. Axes for variables are divided into “bins”, or short intervals, and intensities for each bin are the statistical variables that are analyzed so as to identify relevant spectral features that will distinguish different data sets. After grouping data sets according to their differences, hopefully with the result being that different classes are very distinct, one then invokes one or more approaches for identifying and quantifying the many metabolite concentrations prominent for each group [70,71]. In the second approach to NMR spectral analysis, known as *targeted profiling* [1,72], the first job is identifying and quantifying each metabolite in every NMR spectrum, so that metabolite concentrations are the variables, and the next step can be to use “omics”-based statistical methods to search for meaningful differences among them. In targeted profiling one must have *a priori* knowledge of each metabolite’s complete NMR spectrum, which means knowing the spectral location and relative signal intensity for every ^1^H in each metabolite. The advantages of each method have been compared [72,73]. With chemometrics one makes no assumptions about the identity and quantity of metabolites in the spectra, and, with statistical significance, one separates the spectra into different groups. This is a very appropriate and efficient approach to large data samples where metabolite compositions and quantities might be very different. Thus chemometrics is more easily performed, but its results are less easily translated into harder into clinical or mechanistic significance. In targeted profiling one quantifies specific metabolites and distinguishes spectra based on these. Thus targeted profiling is tedious and more slowly accomplished. In situations where the chemical composition of the sample is known, quantifications use information about the entire shape of a metabolite peak, and, depending on spectral circumstances, will provide more accurate or less accurate metabolite quantifications. There are different approaches to targeted profiling. One approach that has been used for many years is the Linear Combination Model (LC Model), where one finds the best fit to the shape of an NMR spectrum by assuming that it is a linear combination of known spectral lineshapes, each being from a particular metabolite [74]. Interactive fitting via computer software is a more modern method [72].

The basic principle behind “omics” methods, which were made practical by the evolution of fast, powerful computers with large data storage capacity, is its being based on a geometrical approach. One copes with having a large number of biomarkers or predictors for one subject by considering the entire set of numbers as the coordinates of a single point in a multidimensional space. So, if one has 200 different metabolites measured for one biological sample, or gene expression quantifications for 2000 genes from one sample, for metabolomic analyses that sample is represented by a single point in a 200 dimensional space for metabolomics, while for genomic analysis it is represented by a single point in a 2000 dimensional space. If in the multidimensional space one plots data sets for two different groups, for example from two groups of patients where each group had a different treatment for the same condition, then the distribution of points for each of the two groups will, within statistical error, overlap in every single plane or line that one passes through the space. If however, a plane can be found where projections to the plane for one treatment group has a cluster that is significantly separated from a cluster in the other treatment group, then a difference exists that can be modeled by the acquisition of a lot more data. The general process here is known as SIMCA, or Soft Independent Modeling of Class Analogy. And orthogonal directions through the point distributions where variance is maximal are known as Principal Component Axes. Usually the number of axes that will sustain variance differences between two groups is very much less than the number of biomarkers. Often two or three principal component axes are sufficient, but even if the number is larger, the dimension of the space where variations appear is much less than the number of biomarkers, 200 or 2000 in the examples we cited.

There are two general types of pattern recognition algorithms in multivariate statistical analyses: unsupervised and supervised. The most common unsupervised method, Principal Component Analysis (PCA) is usually employed first even if one goes on to complex algorithms with supervised analyses. In unsupervised analyses all variables are treated as random, even though some variables might be strongly dependent on others. In supervised analyses one uses prior information to make two groups of variables: predictors, usually denoted as X, and response variables, usually denoted by Y. The point is to test how data in X determine the behavior of data in Y. Many different algorithms are available, with common ones being PLS-DA (Projection to Least Squares-Discriminant Analyses; often with the words before “DA” being “Projection to Latent Systems”), DPLS (Discriminant Partial Least Squares), and more.

For all algorithms two-dimensional *Scores Plots* are used to display the clusters in planes where the x and y axes are Principal Component axes, and the (x,y) coordinates of a data point comes from projecting the multidimensional vector for that point onto the plane of the chosen Principal Component axes. In the PLS-DA, for example, one algorithm sharpens the PCA clusters and their separations by creating a new Y variable for each data class [75]. The Scores Plots in the PCA and PLS-DA analyses have corresponding *Loadings Plots* that identify the metabolites that contributed most to separations in the Scores Plot. Like in the Scores Plot, points plotted on the Loadings Plot come from projecting multidimensional vectors onto the plane of chosen Principal Component axes. Each point on a Loadings Plot corresponds to a single metabolite, because the vectors projected onto the chosen plane are the component vectors along each dimension that added to make the single vector that represents the full data set. As an example, an NMR data set of fifty metabolites would contribute one point to a Scores Plot, and fifty points to a Loadings Plot. The construction of a model via SIMCA methods requires the acquisition of training data sets that define the geometrical attributes of different classes in the multidimensional space. When different classes have large separations in a Principal Component subspace, the model can be used to predict the likelihood that new data will belong to a particular class. A recent T-Cell study provided a good example [76].

Recent advances in ^1^H NMR data acquisition methods have led to more accurate quantifications of NMR-detectable metabolites. Of special note is the refined technique of *Two-Dimensional* *^1^H J-resolved (JRES) NMR Spectroscopy* [77,78]. J-coupling, which arises from the magnetic field property of particles that have spin, is the indirect coupling between two chemically bound nuclear spins that occurs via the electrons of their chemical bond. In 2D JRES NMR Spectroscopy, the two dimensions into which metabolite signals are separated are: one by chemical shift (usually indicated in ppm units), and a second one by the magnitude of the J-coupling to neighboring protons (usually in units of Hertz). Analogous to when a bar magnet is placed in a magnetic field, a spin ½ nucleus such as ^1^H or ^13^C can exist in either a low energy or high energy state, depending on whether it is aligned with or against the main (or applied) magnetic field, which is constant for a given spectrometer, with the field strength being somewhere between 7 and 22 Tesla. Which energy state that a nucleus, a proton for example, is in influences, to a very small but measurable degree, the magnetic field experienced by the electrons responsible for chemical bonds, and ultimately by adjacent protons. For the simple case of two protons separated by three bonds, each proton resonance, instead of being a singlet peak, will be split into two peaks, *i.e*., into a doublet, due to the fact that the other proton can be in one of two states. The separation in Hertz between the two signals in the doublet is referred to as the J-coupling. It is independent of the strength of the applied magnetic field, because it is determined only by the difference in the magnetic field at one nucleus that is experienced by an orientation change in another nucleus. J-coupling can exist between ^1^H nuclei on adjacent carbons, for example between the methyl protons on lactate and the ^1^H proton on the center carbon (C2), or between ^1^H nuclei and adjacent or nearby ^13^C nuclei. The ^1^H–^1^H J-coupling in lactate is approximately 7 Hz, while the ^1^H–^13^C J-coupling between the lactate’s methyl protons (on C3) and ^13^C in [3-^13^C] lactate is approximately 128 Hz. For the rest of this discussion we will be referring only to ^1^H-^1^H J-coupling.

**Figure 5:**
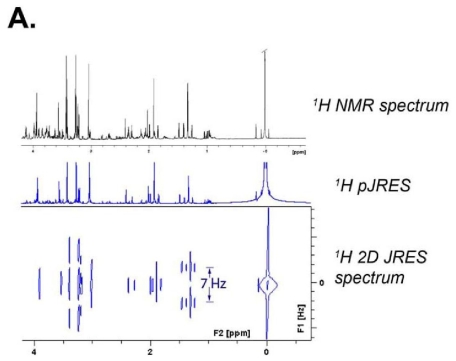

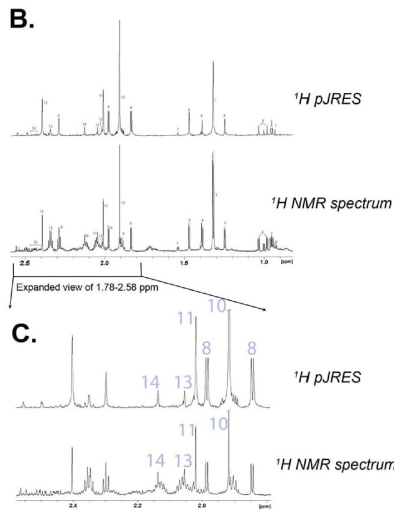
Typical 900 MHz NMR spectra for 2D ^1^H JRES and 1D ^1^H pJRES spectroscopy. (**A**) The ^1^H lactate resonance at 1.33 ppm, which comes from the methyl group on lactate’s middle carbon, provides a good example of the differences that occur in JRES spectroscopy. Because of ^1^H-^1^H J-coupling between methyl ^1^H nuclei and the ^1^H atom on the other side of the methyl group, the ^1^H lactate doublet at 1.33, which has its two peaks separated by 7 Hz or 0.008 ppm, as can be seen in the 2D plot and the black 1D standard spectrum at the top of the figure. In the downwards projection of the 2D plot onto the x-axis, which creates the 1D pJRES spectrum, the 7 Hz doublet merges to form a sharp singlet. There is no lateral dispersion of a nucleus’ NMR signal when one projects the 2D spectrum down onto the ppm axis, because the J-coupling data were aligned vertically. This produces 1D ^1^H NMR spectra in which metabolites have less overlap with each other; (**B**) A comparison is provided between a standard 1D ^1^H spectrum (bottom of the two spectra) and a 1D ^1^H pJRES spectrum (upper of the two spectra). The numbers refer as follows to the metabolites: 1. Isoleucine; 2. Leucine; 3. Valine; 4. Lactate ^13^C; 5. Lactate; 6. Alanine; 7. Alanine ^13^C; 8. Acetate ^13^C; 9. GABA; 10. Acetate; 11. NAA; 12. NAAG; 13. Glutamate; 14. Glutamate/Glutamine; 15. Succinate; 16. Glutamine. The baseline is clearly much flatter, and ripples caused by ^1^H-^1^H J-coupling are very much reduced. Glutamate and glutamine (peaks 13 and 14), which are difficult to quantify in normal 1D spectra, are collapsed into sharp peaks in 1D ^1^H JRES Spectroscopy. Note that when the methyl carbon of lactate is a ^13^C nucleus, one still has the ^1^H-^13^C J-coupling that produces the satellite lactate peaks approximately 0.07 ppm to either side of 1.33 ppm. The ^1^H-^13^C coupling is not collapsed with this type of JRES spectroscopy; (**C**) An expanded view of the glutamate-glutamine region permits better appreciation of the improvement created by JRES Spectroscopy.

The phenomenon of J-coupling is used to separate the ^1^H signals into a second dimension in the 2D JRES experiment, as illustrated in Figure 5. In Figure 5 it is also apparent that the 2D separation of the resonances helps resolve signals that overlap in normal 1D ^1^H spectra. For metabolomics studies, a 2D ^1^H JRES spectrum can be fit directly. The choice of NMR data acquisition parameters for a 2D ^1^H JRES spectrum permits the extraction of a 1D ^1^H spectrum from the 2D dataset in which the J-couplings have been removed. This is because the 1D ^1^H spectrum is obtained by projecting the 2D data straight down onto the horizontal ^1^H axis, and in the 2D data set, the data in the J-coupling dimension are vertically aligned. Thus for any particular ^1^H resonance, data for all J-couplings appear in the projected 1D JRES spectrum at the same ppm. Removal of J-coupling information from 1D ^1^H spectra in this way reduces the overlap of resonance peaks from different metabolites, and allows more accurate metabolite quantifications. Improvements in the identification and quantification of glutamate and glutamine are particularly impressive, as seen in Figure 5 and Figure 4. Other impressive comparisons and elucidations can be found in papers by Viant *et al* [79] and Ludwig and Viant [78]. The notation 1D ^1^H pJRES, which is popular among certain authors, is used to refer to the ^1^H spectrum produced by projecting a 2D ^1^H JRES spectrum onto the ^1^H axis. The 2D ^1^H JRES and 1D ^1^H pJRES approaches are likely to become widely used, as such data can be recorded quickly (~20 min), and 1D and 2D spectra are easy to process and analyze.

Other 2D NMR methods that have been used in metabolomics studies include ^1^H-TOCSY [80] and ^1^H-^13^C HSQC experiments [81,82]. An advanced 2D NMR technique, known as Targeted Projection NMR Spectroscopy [70] can be used when overlapping signals hamper analysis of TOCSY and HSQC spectra. In this method, a “tilted” 2D projection of a hybrid 3D TOCSY-HSQC spectrum is recorded. The projection can be tailored to resolve specific overlapping sets of resonances [83]. A highly cited 2008 publication by Gowda and colleagues reviewed many NMR experiments available to develop diagnoses from metabolic profiles [84].

## 6. Brain Metabolomics and Oxidative Stress

### 6.1. Schizophrenia

Schizophrenia, a complex mental disorder of variable onset that usually develops between the ages of 10 and 40, is characterized by cognitive challenges. It is devastating for those afflicted, who can suffer from any or all of several difficulties, such as thinking logically, differentiating between real and unreal experiences, and having normal emotional responses. It is present in approximately 1.1% of all societies regardless of class, ethnicity, religion, or culture, with new cases per year being approximately 1 in 4000, and in the top 10 causes of disability in developed countries [85]. Neurological investigations point to a “disconnect hypothesis” that occurs over a long period within the brain’s ongoing remodeling of synapses [86]. Neurons that loose synaptic connections can undergo apoptosis, a type of noninflammatory cell death that might account for reductions in brain tissue that have been detected in the relevant frontal cortical areas of schizophrenic patients [87,88]. At the molecular level, impaired antioxidant defenses system and oxidative stress have for a long time been known to be part of the pathophysiology. The very recent review by Yao and Reddy [89] discusses many publications providing evidence from biochemistry, proteomics [90], genetics [91], *in vivo* brain imaging, and metabolomics [92,93] that point to derangements in redox signaling. The very comprehensive review by Prabakaran *et al*. used both proteomics and metabolomics of fresh, pre-frontal brain tissue to convincingly identify differences in oxidative stress enzymes and metabolites between schizophrenic and non-schizophrenic patients [92]. Although there is no cure for schizophrenia at this time, substantial improvements from treatments are possible, and approaches to antioxidant therapy, while likely to have an important role, need considerable more research [94].

### 6.2. Neonatal Asphyxia

Research in neonatal asphyxia is a very active area because of some very positive findings in large, recent clinical trials that have studied mild therapeutic hypothermia (brain temperature decrease of 4° C) treatments immediately after birth [95–98]. Some but not all neonates have greatly improved long term neurological outcomes. Mild hypothermia treatments have not been beneficial to the adult brain immediately after traumatic brain injury or stroke [99–101], despite there being substantial benefits to the adult heart from mild hypothermia after cardiac ischemia [102–105]. A metabolomic investigation of urine in neonates who suffered asphyxia found that elevated urinary organic acids were significantly associated with good and poor neurodevelopmental outcome, and that they were related to oxidative stress [106]. In a metabolomics study of urine using ^1^H NMR spectroscopy in a piglet preclinical model of neonatal asphyxia, metabolic variations were found that were associated with hypoxia levels, with group discriminations primarily due to differences in urea, creatinine, malonate, methylguanidine, hydroxyisobutyric acid [107]. In a rodent brain slice model of neonatal ischemia, a metabolomics approach based on ^1^H/^31^P NMR spectroscopy of perchloric acid brain tissue extracts found that after recovery from injurious oxygen-glucose deprivation (OGD), final ATP levels were severely decreased in a normothermia group, but had recovered to equally to control values after mild hypothermia treatments that began either before OGD or a delay time after it. However, cell death at the end of the experiments was decreased in the immediate hypothermia group, but was equally substantially greater with in the normothermia and delayed hypothermia groups. The Scores Plots in the metabolomics PCA and PLS-DA analyses clearly separated the three groups. Potentially important biomarkers in the ^1^H spectra included PCr, ATP and ADP (all of which were resolved thanks to very high spectral resolution), and were supportive of future potential roles for brain metabolomic monitoring during therapeutic hypothermia [108].

### 6.3. Parkinson’s Disease

Parkinson’s disease, whose major signs are tremor, rigidity, and impaired body movement, is a neurodegenerative disorder of unknown cause that affects million of older people, decreases life expectancy and quality, and is expected in this century to surpass cancer as the second most common cause of death among the elderly [109,110]. Oxidative stress has been strongly implicated in its pathophysiology [47]. The development of biomarkers and medications is of great importance, because early diagnosis, which is very difficult from clinical signs alone, can greatly improve patient management. Metabolomic profiling, by identifying blood markers of DNA damage along with reduced uric acid and greatly increased glutathione, has also implied a strong role for oxidative stress [111]. Attempts at combined proteomics/metabolomics have shown substantial promise [112,113].

### 6.4. Traumatic Brain Injury

Traumatic brain injury (TBI) was investigated in a rodent model with high resolution ^1^H NMR metabolomics studies of brain tissue extracts and plasma at 11.7 Tesla [114]. Regional brain metabolite decreases in ascorbate, and changes in other metabolites showed clear evidence of oxidative stress and excitotoxic brain damage. Significant TBI-associated changes in overall spectral patterns were demonstrated in principal components analysis of brain tissue extracts, but no clear effects were discerned in NMR spectra of plasma. In the other two studies of TBI, oxidative stress is demonstrated by a decrease in anti-oxidative reserves (e.g., ascorbate, GSH, and protein sulfhydrls) [115] and increases in excitatory amino acids [116].

## 7. Diabetes and Kidney-Metabolomics and Oxidative Stress

In the early 1990’s it was appreciated that increased oxidative stress is important in the pathophysiology of diabetes, because the protein glycosylation that occurs during long exposures to hyperglycemia leads to free radical production [117–119]. In diabetic patients, increases in oxidative damage to lipids and proteins correlates with the increases in complications of the disease [21,119].

A recent 9.4 Tesla ^1^H-NMR metabolomic investigation of diabetic retinopathy in humans used a chemometric approach with PCA and PLS-DA analysis to show that spectra for vitreous humor samples clearly separated diabetic patients from non-diabetic patients. Among 14 very clearly distinguished metabolites, decreased ascorbate and increased galactitol were the main biomarker changes for diabetic retinopathy, although increased lactate and glucose were found in samples from all diabetics [120]. The decrease in ascorbate was attributed to higher consumption from higher levels of oxidative stress. A recent preclinical study of diabetes used a streptozotocin rat model to study urine and plasma metabolomics with ^1^H NMR at 11.75 Tesla [121]. Spectra were analyzed with chemometrics and profiling that identified 17 metabolites. Plasma TBARS (thiobarbituric acid-reacting substances) were quantified as a measure of oxidative stress. Diabetic rats had altered metabolic pathways and augmented fat degradation products caused by free radicals.

With regard to metabolomic investigations of oxidative stress in the kidney, one human study, in which it was known *a priori* that cyclosporine nephrotoxicity initially occurs in the proximal tubule, somewhat driven by increased formation of oxygen radicals, found that metabolomics measures with ^1^H-NMR and HPLC-MS are sensitive enough to detect toxicity as soon as 4 h after a single oral dose, and could serve as a tool for clinical diagnoses [122]. Several rodent studies used similar metabolomics methods to detect and quantify oxidative stress in the kidney from ischemia/reperfusion injury [123], radiation injury [124], and drug toxicities for acetaminophen [125], cinnabar [126], fenofibrate [127], and ochratoxin [128].

## 8. Liver Metabolomics-Metabolomics and Oxidative Stress

Two very recent liver metabolomics studies provided data on oxidative stress in humans. In the first [129], plasma profiles in patients were studied for three pathological conditions: nonalcoholic fatty liver disease, steatosis, and steatohepatitis. Metabolomic data could separate healthy subjects from those with fatty liver disease with an error rate of approximately 8% and separate those with steatohepatitis from healthy controls with an error rate of 4%. However, metabolomics data could not separate patients with hepatic steatosis from those with steatohepatitis. Marked changes in bile salts and glutathione-related biochemicals were identified in the different group, and a panel of biomarkers was found that can potentially be used to follow response to therapeutic interventions.

In liver transplantation one first causes oxidative stress by inducing ischemia during organ removal from the donor, and then one causes oxidative stress by reoxygenation and perfusion after organ implantation in the recipient. Thus hepatotoxicity in liver transplantation acutely involves considerable oxidative stress. In a very recent human study of 8 liver transplant patients [130], consecutive biopsy findings included that metabolomics time-profiles demonstrated considerable changes in redox-active metabolites up until 18 h postreperfusion, after which there was stabilization. Metabolomics data came from Fourier Transform Ion Cyclotron Resonance Mass Spectrometry (FT-ICR MS) of liver biopsy tissue, and also from microdialysates of extracellular fluid by coulometric electrochemical array detection (CEAD). FT-ICR MS reproducibly detected more than 4,000 peaks, revealing hundreds of significant metabolic differences between pre- and postreperfusion grafts.

In the literature of preclinical experiments there were numerous studies in rodents that showed distinct associations between metabolomic data and experimental manipulations. For example, deficiency of α-tocopheral was studied [131], as were hepato- and nephron-toxicants [132], obesity [133], and acetaminophen toxicity [134].

One important function of the liver is to detoxify poisons that come into an organism, especially via the gastrointestinal system. *Xenobiotics* are chemicals found in organisms that are not normally produced or expected to be present. The high importance of being able to recognize toxins motivated the formation by certain leaders in metabonomics research of *The Consortium for Metabonomic Toxicology* (COMET), which is between five major pharmaceutical companies and The Imperial College of London [135]. This group has assessed methodologies and used rodent urine and blood to generate a ^1^H NMR metabonomic database for use in evaluating and dealing with xenobiotic toxicity.

## 9. Cardiac and Vascular Metabolomics and Oxidative Stress

Investigations with human tissues and fluids included a LC-MS (liquid chromatography and mass spectroscopy) metabolomics study of plasma lipids that was done for 14 patients having documented silent myocardial ischemia (SMI), as compared to 25 matched control patients [136]. Scores Plots in the multivariate statistic analysis showed a clear separation between the two studied groups. Plasma concentrations of four phospholipids had a tight association with SMI, with 1-linoleoylglycerophosphocholine (C18:2) being significantly decreased in the SMI group, suggesting that such measurements might facilitate early diagnosis. A careful metabolomics study of hypoxia in humans [137], motivated by the deleterious roles hypoxia plays in cardiovascular disorders and numerous diseases, looked for evidence of oxidative stress, and also for evidence that HIF, hypoxia-inducible transcription factor, is activated *in vivo* in humans during hypoxia. The group used a hypobaric chamber to expose fourteen human subjects to normoxia corresponding to an altitude of 1600 meters, and to hypoxia corresponding to an altitude of 4300 m (alveolar PO_2_ ≈ 48 mm Hg). HIF-1 DNA binding and HIF-1α protein were evaluated in circulating leukocytes, and several markers of oxidative stress were among 47 metabolites studied with ^1^H NMR plasma metabolomics. Circulating glutathione was reduced by 35% (*p* = 0.001), and lactate and succinate were increased by 29 and 158%, respectively (*p* = 0.007 and 0.001), as were urinary 15-F2t-isoprostanes (*p* = 0.037), showing that hypoxia causes substantial increases in plasma and urinary markers of oxidative stress. Another metabolomics study in humans [138] used LC-MS to look for oxidative stress in the etiology of abdominal aortic aneurysms (AAA), the 13th leading cause of death in the United States with up to 9,000 deaths annually. An AAA is a permanent, localized aortic dilation greater than 3 cm in diameter. AAA enlargement occurs irregularly, and pathology of the involved vascular tissue has been associated previously with oxidative stress, chronic inflammation, elastin fragmentation, apoptosis, loss of extracellular matrix, neovascularization, and a depletion of smooth muscle cells. Metabolites were compared that came from different portions of human aortic aneurysms--luminal and abluminal layers, aneurysm walls, and healthy walls. Compounds related to inflammation and oxidative stress were found, as was a possible role of fatty acid amides. Some metabolites (e.g., hippuric acid) had not been previously associated with such aneurysms.

Several recent animal studies have used metabolomics to investigate oxidative stress in cardiovascular systems. Complementary to the human data just cited are proteomic and metabolomic analyses of atherosclerotic vessels from apolipoprotein E-deficient mice that revealed alterations in inflammation, oxidative stress, and energy metabolism [139] Oxidative stress in atherogenesis was apparent in that study from the upregulation of numerous antioxidant proteins the detection of ROS protein damage, and the increase in albumin degradation. Damage was found in enzymes susceptible to free radical inactivation, including aconitase, cytochrome C reductase, and the Rieske protein of ubiquinol. Such damage can release free iron, thereby promoting subsequent formation of hydroxyl radicals, and perpetuating oxidative stress, as explained early in the review. Proteomic and metabolomics measures were made in a dog model for congestive heart failure and ventricular tachycardia, with the heart paced at 240 beats per min for 24 h in one group, and for 2 weeks in another [140]. Oxidant stress was evident in the 24 h group from the substantial decrease in antioxidant proteins, including SOD and peroxiredoxin. Heat shock protein increases and other protein changes were analyzed, as was the metabolic stress related to congestive heart failure (CHF). That study provided insights to the arrhythmogenic atrial remodeling that results from such physiological stress. In an initial study by others of mice deficient in protein kinase C delta [141], proteomic and metabolomics analysis of smooth muscle cells demonstrated that loss of PKC delta improved the cellular redox state by elevating GSH levels, and thus provided protection against oxidative stress-induced cell death. However, in a second study with mice deficient in protein kinase C delta [28], they showed that the loss of PKC delta caused the loss of protection provided by ischemic preconditioning, because transient increases in reactive oxygen species cause preconditioning, while antioxidants block the beneficial effects of preconditioning. The two studies, besides adding useful specific biochemical information, provide a good example of a general point made earlier, that antioxidant augmentation is not good in situations where it impairs host defenses.

## 10. Cancer Metabolomics and Oxidative Stress

Metabolic derangements and adaptations by cancers have recently been carefully reviewed [142], with two prominent features being: (1) excess glucose metabolism and lactate production with avoidance of the TCA cycle, even in the presence of substantial oxygen availability [Warburg Effect]; and (2) mitochondrial malfunction with augmented ROS production. The role of ROS in tumor progression is controversial, because increased ROS can serve what might appear to be opposite functions, namely induction of apoptosis, or augmentation of mutagenesis and tumor proliferation. Apoptosis can also be induced by radiation therapy of tumors. Indeed, radiation toxicity is attributed to the increased oxidative stress triggered by direct ionization of biological polymers and the generation of reactive oxygen species via radiolysis of water, which produces hydroxyl radicals [143]. In radiation therapy, however, there are adaptive responses. A study of 22 metabolites from healthy human leukocytes that had radiation exposures of 2 Gy, 4 Gy and 8 Gy found that relative to control data at 0 Gy, metabolites were substantially depleted at 2 Gy and 8 Gy, but substantially upregulated at 4 Gy, with many at values greater than control. A recent review [144] discussed metabolomics and oxidative stress in pheochromocytomas, endocrine cell tumors that come from chromaffin cells in the adrenal medulla and can produce enormous amounts of catecholamines.

## 11. Conclusions

A wide variety of publications were found that make important clinical and mechanistic associations between oxidative stress and specific metabolites, metabolite groups, and metabolomics analyses. Table A1 provides a compendium of associations between pathological situations with oxidative stress, and metabolites identified in particular studies, all of which are cited in the list of references. Included in the table are references to six publications [145–150] that had valuable information, but matched more than one the categories we established, and were not referenced earlier.

Complex relationships connect oxidative stress to many physiological processes. It is hoped that by developing multivariate analyses techniques it will be able to discern characteristics of metabolite ensembles that distinguish different physiological processes, and in so doing, be able to learn key mechanistic aspects and also develop useful interventions. The goal of studying the metabolomics of oxidative stress is to develop methods that serve as a tool for diagnoses, treatment guidance, and drug discovery. The rapid pace of research in this area gives hope for such progress.

## Appendix

**Table A1:** Metabolomic studies in different diseases associated with oxidative stress.

Pathology	Change of metabolites	Samples	Analytical technique	Ref.	Year
**CNS**					
Schizophrenia	Taurine ↑; Glutathione	Prefrontal cortex tissue from patients	^1^H NMR	[92]	2004
Schizophrenia	Glucose ↑; Acetate ↓; Alanine; Glutamine	CSF from human	^1^H NMR	[145]	2006
Schizophrenia	Phosphotidylethanolamine ↓ Phosphotidylcholine ↓	Lipid extracts of patient’s plasma	HPLC	[146]	2007
Parkinson’s disease	Uric acid ↓; GSSG ↑	Plasma from patients	LCECA	[111]	2008
Parkinson’s disease	Urate ↓	Plasma from patients	LCECA	[147]	2009
Trauma Brain Injury	Ascorbate ↓; Glutamate ↓; NAA ↓; Total of PC/GPC ↓	Brain tissue extracts and plasma from rats	^1^H NMR	[114]	2005
Asphyxia	Glutarate ↑; Methylmalonate ↑; 3-hyroxy-butyrate ↑; Orotate ↑	Urine from patients	MS	[106]	2006
Hypoxia-reoxygenati on	Urea; Creatinine; Malonate; Methylguanidine; Hydrooxyisobutyric acid	Urine from piglets 1–4 days old	^1^H NMR	[107]	2010
Oxygen-Glucose deprivation	PCr; ATP ; NAA; Taurine	Rat brain slice extracts	^1^H/^31^P NMR	[108]	2011
**Diabetes and Renal diseases**					
Proliferative diabetic retinopathy	Ascorbate ↓; Galactitol ↓	Vitreous samples from patients	^1^H NMR	[120]	2010
Type 1 diabetes	Glucose ↑; Alanine ↑; Lactate ↑; Ethanol ↑; Acetate ↑; Fumarate ↑	Urine and plasma from rats	^1^H NMR	[148]	2008
Cyclosporine nephrotoxicity	15-F(2t)-isoprostane ↑; Creatinine ↑; Citrate ↓; Hippurate ↑; Phenylalanine	Urine and plasma from human	^1^H NMR HPLC-MS	[122]	2010
Kidney transplantation	Allantoin ↑; Polyunsaturated fatty acids ↓	Kidney tissue and plasma from rats	^1^H NMR	[123]	2005
Radiation injury	Glyoxylate ↑; Threonate ↑; Thymine ↑; Uracil ↑; Citrate ↓; Adipate ↓; Pimelate ↓; Suberate ↓; 2-oxoglutarate ↓	Urine from rats	GC-MS	[124]	2009
Ochratoxin A toxicity	2-oxoglutarate ↓; Citrate ↓; Glucose ↑; Creatinine ↑; Pseudouridine ↑; 5-oxoproline ↑; Myo-inositol ↑	Urine from rats	GC-MS ^1^H NMR LC-MS	[128]	2009
Cisplatin-induced nephrotoxicity	Glucosuria ↑; Nonesterified fatty acids ↑; Triglycerides ↑	Kidney tissue, urine, plasma from rats	^1^H NMR	[149]	2007
**Cardiovascular diseases**					
Hypoxia	Glutathione ↓; Lactate ↑; Succinate↑	Plasma and urine from human	^1^H NMR	[137]	2009
Abdominal aortic aneurysms	Fatty acid amides	Aortas tissue from human	LC-MS	[138]	2011
Atherosclerosis	Alanine ↓; 1-Cys peroxiredoxin (identified in proteomics)	Mouse aortas tissue	^1^H NMR	[139]	2005
H_2_O_2_-induced stress	Alanine ↓; Lactate ↑; Carnitine ↑; Glutathione↓	Mouse C2C12 myotubes	MS	[141]	2004
Doxorubicin cardiotoxicity	Acetate ↑; Succinate ↑	Dog heart tissue extracts	^1^H NMR	[150]	2009
**Hepatic diseases**					
Fatty liver disease Steatohepatitis	Glycocholate↑; Taurocholate↑ Carnitine↑; Butyrylcarnitine↑; Cysteine/Glutathione↓	Plasma from patients	GC-MS	[129]	2011
Acetaminophen-induced hepatotoxicity	Ophthalmate ↓	Mouse liver extracts and serum	CE-TOFMS	[134]	2006
Obesity	Hepatic ratios of PUFA/MUFA ↓ Glutathione ↓	Liver lipid extracts and serum from rat	^1^H NMR	[133]	2006
**Cancer**					
Ionizing radiation	Arginine ↑; Glutamine ↑; Creatine ↑; Proline ↑; GSH↑	Human leukocytes	CE-ESI-MS	[143]	2010

LCECA: Liquid chromatography coupled with eletrochemical coulometric array; GC-MS: Gas chromatography-mass spectrometry; LC-MS: Liquid chromatography-mass spectrometry; CE-TOFMS: Capillary eletrophoresis time-of-flight mass spectrometry; CE-ESI-MS: Capillary electrophoresis mass spectrometry.
